# Factors associated with utilization of quality antenatal care: a secondary data analysis of Rwandan Demographic Health Survey 2020

**DOI:** 10.1186/s12913-022-08169-x

**Published:** 2022-06-22

**Authors:** Quraish Sserwanja, Lilian Nuwabaine, Ghislaine Gatasi, Julius N. Wandabwa, Milton W. Musaba

**Affiliations:** 1 Programmes Department, GOAL, Arkaweet Block 65 House No. 227, Khartoum, Sudan; 2 School of Nursing and Midwifery, Aga Khan University, Kampala, Uganda; 3 Key Laboratory of Environmental Medicine Engineering, School of Public Health, Southeast University, Nanjing, 210009 Jiangsu Province China; 4 Department of Obstetrics and Gynaecology, Busitema University/ Mbale Regional Referral and Teaching Hospital, Mbale, Uganda

**Keywords:** Rwanda, Antenatal Care, Quality, Women, DHS

## Abstract

**Background:**

Over the last decade, progress in reducing maternal mortality in Rwanda has been slow, from 210 deaths per 100,000 live births in 2015 to 203 deaths per 100,000 live births in 2020. Access to quality antenatal care (ANC) can substantially reduce maternal and newborn mortality. Several studies have investigated factors that influence the use of ANC, but information on its quality is limited. Therefore, this study aimed to identify the determinants of quality antenatal care among pregnant women in Rwanda using a nationally representative sample.

**Methods:**

We analyzed secondary data of 6,302 women aged 15–49 years who had given birth five years prior the survey from the Rwanda Demographic and Health Survey (RDHS) of 2020 data. Multistage sampling was used to select RDHS participants. Good quality was considered as having utilized all the ANC components. Multivariable logistic regression was conducted to explore the associated factors using SPSS version 25.

**Results:**

Out of the 6,302 women, 825 (13.1%, 95% CI: 12.4–14.1) utilized all the ANC indicators of good quality ANC); 3,696 (60%, 95% CI: 58.6–61.1) initiated ANC within the first trimester, 2,975 (47.2%, 95% CI: 46.1–48.6) had 4 or more ANC contacts, 16 (0.3%, 95% CI: 0.1–0.4) had 8 or more ANC contacts. Exposure to newspapers/magazines at least once a week (aOR 1.48, 95% CI: 1.09–2.02), lower parity (para1: aOR 6.04, 95% CI: 3.82–9.57) and having been visited by a field worker (aOR 1.47, 95% CI: 1.23–1.76) were associated with more odds of receiving all ANC components. In addition, belonging to smaller households (aOR 1.34, 95% CI: 1.10–1.63), initiating ANC in the first trimester (aOR 1.45, 95% CI: 1.18–1.79) and having had 4 or more ANC contacts (aOR 1.52, 95% CI: 1.25–1.85) were associated with more odds of receiving all ANC components. Working women had lower odds of receiving all ANC components (aOR 0.79, 95% CI: 0.66–0.95).

**Conclusion:**

The utilization of ANC components (13.1%) is low with components such as having at least two tetanus injections (33.6%) and receiving drugs for intestinal parasites (43%) being highly underutilized. Therefore, programs aimed at increasing utilization of ANC components need to prioritize high parity and working women residing in larger households. Promoting use of field health workers, timely initiation and increased frequency of ANC might enhance the quality of care.

**Supplementary Information:**

The online version contains supplementary material available at 10.1186/s12913-022-08169-x.

## Background

According to the 2019 World Health Organization's (WHO) report, over 810 maternal deaths are registered every day, with over 94% occurring in low- and middle-income countries (LMICs) [1]. Most of these deaths could be prevented or significantly reduced through interventions such as receiving quality antenatal care (ANC), skilled birth attendance (SBA) and post-natal care (PNC) [2, 3]. The WHO’s latest ANC recommendations emphasize the importance of every pregnant woman receiving prompt, appropriate, and high-quality care throughout her pregnancy, childbirth, and postpartum period [4, 5].

Antenatal care (ANC) can be described as care given to pregnant women by professional health care providers to ensure the best health outcomes for both mothers and babies during pregnancy and child birth [6]. It also serves as an entry point to the utilization of maternal care services. Evidence shows that women that receive quality services during ANC develop confidence in the maternal care services, are more likely to deliver under the care of a skilled birth attendant, and also seek early postnatal care (PNC) [2, 5]. The aim of ANC is to help women sustain normal pregnancies through early identification of preexisting conditions, identifying complications that arise during childbirth, and promotion of well-being [2, 7]. Currently, the WHO guidelines recommend at least eight contacts, with the first one occurring within the first 12 weeks of gestation [8].

In sub-Saharan Africa (SSA), several countries have reported a significant improvement in the utilisation of ANC services by pregnant women, yet the maternal and neonatal morbidity and mortality has remained unacceptably high [7, 9]. Adequate and quality ANC has been documented to contribute towards reduction of maternal and neonatal mortality. Tiruye et al. showed that attending at least one ANC contact can decrease the risk of neonatal death by 42% [10]. However, SSA regions such as East Africa continue to share some of the poorest ANC utilization statistics and a significant share of global maternal and newborn mortality [10, 11]. This may be related to the quality of ANC care that is provided, which has been criticized in several studies from SSA [12]. Recently published data have documented low ANC utilization in East Africa with overall quality of ANC at 11.6%, utilization of at least 4 contacts at 56.4% and use of skilled ANC providers at 78.7% [11, 13]. Countries like Ethiopia have reported the lowest quality of ANC at 5.6% [13]. With the highest proportion of at least four ANC contacts in East Africa observed in Uganda at 61%, this shows the need to prioritize ANC programs in the region to ensure progress in the reduction of maternal and neonatal mortalities [11].

Rwanda was among the countries that achieved the Millennium Development Goals (MDG) 4 and 5 [14, 15] and also registered reduction in the maternal mortality ratio (MMR) from 750 deaths per 100,000 live births in 2005 to 210 deaths per 100,000 live births in 2015 [16]. However, very slow progress has been registered between 2015 and 2020 from 210 deaths per 100,000 live births to 203 deaths per 100,000 live births [16]. Furthermore, according to the latest Rwanda Demographic and Health Survey (RDHS), less than half of all the women are able to utilise more than four ANC contacts, yet over 94% deliver under the care of a SBA [16]. This is further supported by a regional paper that shows Rwanda to have the lowest optimal ANC utilization proportion [11]. In order to ensure successful implementation of the latest WHO ANC guidelines that recommend at least eight contacts for every pregnant woman and progress towards reduction of the MMR to less than 70 per 100,000 live births, there is need to explore the different factors such as quality of ANC that might affect utilisation of ANC [4, 8, 17]. In addition to affecting pregnancy outcomes, inadequate quality of care might discourage women from utilizing ANC and other maternal healthcare services [12, 18]. Although several studies that have looked at ANC in Rwanda, no post MDG era national study has been able to extensively evaluate ANC in the country with a focus on the utilization of ANC components and also considering the latest WHO ANC guidelines. Therefore, this study aimed mainly to identify the factors associated with utilization of ANC components among pregnant women in Rwanda using a nationally representative sample of 2020 RDHS.

## Methods

### Setting

Rwanda located in central-eastern part of Africa is a low-income country with a population of about 12 million people [16, 19]. Rwanda’s public health system comprises of national referral hospitals as the highest level of care followed by provincial hospitals, district hospitals, health centers, and health posts [20, 21]. Community health workers (CHWs) who are over 45,000 provide health services at the village level [20, 22]. These CHWs provide the first line of basic health services with each village having a male–female CHW pair [20]. Rwanda has a universal, community-based health insurance program that has a household subscription and co-payments at the time of care and all citizens are eligible to enroll into it [20, 23]. Community-Based Health Insurance (CBHI) is purchased by about 86% of households [24].

### Study design

Cross sectional study to analyze secondary data.

### Study sampling and participants

The 2019–20 Rwanda Demographic Survey (RDHS) was used for this analysis. Data collection started in November 2019 and ended in July 2020 taking longer than expected due to the COVID-19 pandemic restrictions [16]. The Rwanda National Ethics Committee (RNEC) and the ICF Institutional Review Board reviewed and approved the survey protocol [16]. The2019-20 RDHS employed a two-stage sample design with the first stage involving sample points (clusters) selection consisting of enumeration areas (EAs) leading to 500 clusters being selected (112 in urban areas and 388 in rural areas) [16]. The second stage involved systematic sampling of households in all the selected EAs leading to a total of 13,005 households [16]. The RDHS used five questionnaires that included: the household, the woman’s, the man’s, the biomarker, and the fieldworker questionnaires. The data used in this analysis were from the household and the woman’s questionnaires.

Women aged 15–49 years who were either permanent residents of the selected households or visitors who stayed in the household the night before the survey were eligible to be interviewed. Out of the total 13,005 households that were selected for the survey, 12,951 were occupied and 12,949 were successfully interviewed leading to 100% response rate of 100.0% [16]. This study included women aged 15–49 years who were in need of ANC having had childbirth within five years preceding the survey. For those with more than one birth, the latest birth was considered. Among the interviewed households,14,675 women aged 15–49 were eligible to be interviewed and 14,634 women were successfully interviewed leading to a 99.7% response rate [16]. Out of the 14,634 successfully interviewed women, a weighted sample of 6,302 women had given birth within the last five years preceding the survey as shown in the supplementary file 1.

### Variables

#### Dependent variables

The primary outcome variable was complete utilization of ANC components available in the RDHS women dataset that included: having blood pressure measurement, urine and blood samples being taken, being given iron tablets/syrups and intestinal parasite drugs and having had at least two tetanus injections [25–27]. Complete utilization of all the six ANC components was considered a proxy for having received good quality ANC and was coded 1 while inadequate quality was coded zero [25, 28].

The secondary outcomes were timing of ANC initiation and frequency of ANC visits. As recommended by the latest WHO guidelines, early ANC initiation was considered as initiation within the first trimester coded as one and initiation after first trimester coded as zero [27]. Adequate ANC frequency was considered as 4 and more contacts and coded as one and less than 4 contacts coded as zero [2, 6]. However, sensitivity analysis was done using 8 or more contacts as a measure of adequate ANC frequency recommended by the latest WHO guidelines [27].

#### Independent variables

This study included determinants of ANC initiation timing, frequency and quality based on evidence from available literature and data [6, 16, 25, 28]. Twenty explanatory variables were used in this study as shown in Table 1.

**Table 1:** Categorization of independent variables

Variable	Categorization	Description
Exposure to newspapers or magazines	Not at all Less than once a week’ At least once a week	RDHS had four original categories (not at all, less than once a week, at least once a week and almost every day). However, after data collection, no woman reported almost every day
Exposure to radio	Not at all Less than once a week’ At least once a week	RDHS had four original categories (not at all, less than once a week, at least once a week and almost every day). However, after data collection, no woman reported almost every day
Exposure to television (TV)	Not at all Less than once a week’ At least once a week	RDHS had four original categories (not at all, less than once a week, at least once a week and almost every day). However, after data collection, no woman reported almost every day
Access to internet	Yes and no	
Age	15 to 19 years, 20 to 34 years, and 35 to 49 years	
Residence	Urban and Rural	
Region	North, East, South, West and Kigali	
Household size	Less than 6 and above 6	Based on the dataset average of 5.2
Parity	1, 2–4, 5, and above	
Level of education	No education, primary, secondary, and tertiary	
Working status	Yes and no	
Wealth index	Richest, richer, middle, poorer, and poorest quintiles	The RDHS collected data on household asset ownership and calculated wealth index using Principal Component Analysis [16]
Having health insurance	Yes and no	
Having been visited by a field health worker within the last 12 months	Yes and no	
Problems seeking permission and distance to health facility	No big problems and big problems	RDHS had three original categories (no problem, no big problem and big problem) however, after data collection, no woman reported no problem
Problems with distance to nearest health facility	No big problems and big problems	RDHS had three original categories (no problem, no big problem and big problem) however, after data collection, no woman reported no problem
Marital status	Married and not married	Married included both formal and informal unions
Place of ANC	Private and public health facilities	Private facilities included polyclinics, clinics, and dispensaries while public included referral and district hospitals, health centers, posts, and outreaches. These were combined due to the limited numbers in each sub-category
ANC initiation timing	Within first trimester and after first trimester	
ANC frequency	4 and above contacts and less than 4 contacts	

### Statistical analysis

RDHS sample weights were used through the analysis to account for the unequal probability sampling in different strata [29] and to ensure representativeness of the findings [30]. In order to account for the multistage sample design inherent in the DHS dataset and to avoid any effect of the study design on the results hence ensuring accurate and reliable results, SPSS version 25.0 statistical software complex samples package was used. The complex samples’ package the analysis plan incorporated the sample individual weight, strata for sampling errors/design, and cluster number used in the RDHS which accounted for the multistage sample design inherent in the RDHS dataset [31–33]. Furthermore, use of weights enables making statistical inference at the population level while incorporating strata and cluster ensures getting correct standard error. Bivariable logistic regression was done to assess the association of each independent variable with each outcome and crude odds ratio (COR), 95% confidence interval (CI) and *p*-values are presented. Independent variables found significant at bivariable level with *p*-values less than 0.25 were added in the multivariable logistic regression model. Adjusted odds ratios (AOR), 95% Confidence Intervals (CI) and *p*-values were calculated with statistical significance level set at *p*-value < 0.05 [34]. All variables in the model were assessed for collinearity, which was considered present if the variables had a variance inflation factor (VIF) greater than 3. Sensitivity analysis was done with 8 or more ANC contacts as the outcome.

## Results

A total of 6,302 women were included in the analysis (Table 2). Majority of the women resided in rural areas (82.2%), had primary education (64.6%), had no exposure to newspapers (80.5%), no internet access (89.3%), had health insurance (81.1%), were married (80.8%) and were working (75.7%). Out of the 6,302 women, 3,696 (60%, 95% CI: 58.6–61.1) initiated ANC within the first trimester, 2,975 (47.2%, 95% CI: 46.1–48.6) had 4 or more ANC contacts, 16 (0.3%, 95% CI: 0.1–0.4) had 8 or more ANC contacts and 825 (13.1%, 95% CI: 12.4–14.1) utilized all the ANC components (good quality ANC). Details of the ANC components are shown in Table 3.

**Table 2:** Socio-demographic characteristics of women in Rwanda as per the 2020 RDHS

Characteristics	*N* = 6,302	%
**Age**		
35 to 49	2207	35.0
20 to 34	3970	63.0
15 to 19	125	2.0
**Education Level**		
No Education	698	11.1
Primary Education	4071	64.5
Secondary Education	1258	20.0
Tertiary	275	4.4
**Exposure to newspapers/magazines**		
No	5070	80.5
Less than once a week	854	13.5
At least once a week	378	6.0
**Exposure to radio**		
No	1448	23.0
Less than once a week	1202	19.0
At least once a week	3652	58.0
**Exposure to TV**		
No	3753	59.5
Less than once a week	1518	24.1
At least once a week	1031	16.4
**Internet access**		
No	5626	89.3
Yes	676	10.7
**Parity**		
1	1587	25.2
2–4	3550	56.3
5 and above	1165	18.5
**Marital**		
Not married	1208	19.2
Married	5094	80.8
**Has health insurance**		
No	1194	18.9
Yes	5108	81.1
**Visited by a fieldworker**		
No	3994	63.4
Yes	2307	36.6
**Working status**		
Not working	1532	24.3
Working	4770	75.7
**Permission to access healthcare**		
Big problem	222	3.5
Not big problem	6080	96.5
**Distance to health facility**		
Big problem	1461	23.2
Not big problem	4841	76.8
**Residence**		
Rural	5179	82.2
Urban	1123	17.8
**Region**		
North	1004	15.9
East	1702	27.0
West	1425	22.6
South	1305	20.7
Kigali	866	13.7
**Household Size**		
6 and above	2357	37.4
Less than 6	3945	62.6
**Wealth Index**		
Poorest	1448	23.0
Poorer	1217	19.3
Middle	1224	19.4
Richer	1234	19.6
Richest	1178	18.7

Bold: independent variables included in the study

**Table 3:** Antenatal care components

Service	Frequency *N* = 6,302	%
At least 2 TT injections	2117	33.6
Blood pressure measured	5506	87.4
Blood sample taken	6160	95.5
Urine sample taken	5208	82.6
Given iron tablets /syrup	5081	80.6
Given drugs for intestinal parasites	2709	43.0
Received all components	825	13.1

*TT* Tetanus toxoid, Bold: ANC components that were included in the analysis

### Factors associated with utilization of all ANC components

Exposure to newspapers/magazines at least once a week (aOR 1.48, 95% CI: 1.09–2.02), lower parity (para1: aOR 6.04, 95% CI: 3.82–9.57), having been visited by a field worker (aOR 1.47, 95% CI: 1.23–1.76) and belonging to smaller households (aOR 1.34, 95% CI: 1.10–1.63) were associated with more odds of utilizing all ANC components. Furthermore, initiating ANC in the first trimester (aOR 1.45, 95% CI: 1.18–1.79) and having had 4 or more ANC contacts (aOR 1.52, 95% CI: 1.25–1.85) were associated with more odds of utilizing all ANC components. Working women had lower odds of utilizing all ANC components (aOR 0.79, 95% CI: 0.66–0.95) as shown in Table 4. Factors associated with secondary outcomes (timing of ANC initiation and ANC frequency) are shown in supplementary file 2.

**Table 4:** Factors associated with utilization of all ANC components as per RDHS 2019–20

Characteristics	ANC quality Crude odds ratio cOR (95% CI)	*P*-value	ANC quality Adjusted model aOR (95% CI)
**Age**			
35 to 49	1		1
20 to 34	**2.13 (1.78–2.56)**	< 0.001	1.04 (0.82–1.30)
15 to 19	**2.54 (1.52–4.26)**	< 0.001	0.80 (0.46–1.40)
**Education Level**			
No Education	1		1
Primary Education	1.13 (0.86–1.48)	0.383	0.76 (0.56–1.02)
Secondary Education	**2.01 (1.49–2.70)**	< 0.001	0.90 (0.62–1.28)
Tertiary	**1.95 (1.31–2.88)**	0.001	0.93 (0.53–1.62)
**Exposure to newspapers/magazines**			
No	1		1
Less than once a week	**1.40 (1.07–1.82)**	< 0.001	1.13 (0.86–1.48)
At least once a week	**1.93 (1.46–2.54)**	0.014	**1.48 (1.09–2.02)**
**Exposure to radio**			
No	1		1
Less than once a week	**1.30 (1.01–1.67)**	0.047	1.18 (0.90–1.56)
At least once a week	**1.31 (1.07–1.60)**	0.009	0.97 (0.77–1.23)
**Exposure to TV**			
No	1		**1**
Less than once a week	1.21 (0.99–1.47)	0.065	1.05 (0.84–1.30)
At least once a week	**1.36 (1.10–1.70)**	0.007	1.35 (0.99–1.85)
**Internet access**			
No	**1**		**1**
Yes	**1.52 (1.25–1.86)**	< 0.001	0.80 (0.58–1.11)
**Parity**			
5 and above	1		**1**
2–4	**3.53 (2.44–5.12)**	< 0.001	**2.65 (1.74–4.02)**
Less than 2	**8.42 (5.80–12.23)**	< 0.001	**6.04 (3.82–9.57)**
**Marital**			
Not married	1		**1**
Married	**0.76 (0.62–0.92)**	0.005	0.86 (0.69–1.06)
**Has health insurance**			
No	1		**1**
Yes	**1.41 (1.14–1.74)**	0.001	1.02 (0.81–1.29)
**Visited by a fieldworker**			
No	1		**1**
Yes	**1.49 (1.25–1.78)**	< 0.001	**1.47 (1.23–1.76)**
**Working status**			
Not working	1		**1**
Working	**0.70 (0.59–0.83)**	< 0.001	**0.79 (0.66–0.95)**
**Permission to access healthcare**			
Big problem	1		**-**
Not big problem	1.23 (0.81–1.87)	0.334	**-**
**Distance to health facility**			
Big problem	1		**1**
Not big problem	**1.37 (1.11–1.70)**	0.004	1.21 (0.96–1.53)
**Residence**			
Rural	1		1
Urban	**1.21 (1.01–1.45)**	0.039	1.10 (0.85–1.41)
**Region**			
North	1		1
East	0.84 (0.61–1.17)	0.300	0.83 (0.59–1.15)
West	0.90 (0.67–1.21)	0.483	0.99 (0.73–1.34)
South	1.19 (0.90–1.57)	0.224	1.10 (0.83–1.47)
Kigali	1.01 (0.74–1.38)	0.955	0.84 (0.59–1.20)
**Household Size**			
6 and above	1		1
Less than 6	**2.12 (1.77–2.53)**	< 0.001	**1.34 (1.10–1.63)**
**Wealth Index**			
Poorest	1		1
Poorer	1.09 (0.85–1.40)	0.519	1.07 (0.82–1.41)
Middle	0.96 (0.73–1.25)	0.738	0.99 (0.73–1.34)
Richer	**1.41 (1.10–1.81)**	0.007	1.23 (0.93–1.63)
Richest	1.26 (0.98–1.61)	0.068	0.88 (0.59–1.30)
**ANC timing**			
After first trimester	1		1
Within first trimester	**2.09 (1.76–2.48)**	< 0.001	**1.45 (1.18–1.79)**
**ANC frequency**			
Less than 4	1		1
4 and above	**2.09 (1.77–2.46**)	< 0.001	**1.52 (1.25–1.85)**
**ANC facility**			
Private	1		1
Public	**2.28 (1.43–3.62)**	0.001	1.65 (0.99–2.75)

Bold: significant at < 0.05, adjusted analysis for age, education level, exposure to newspapers, radio and TV, internet access, parity, marital status, health insurance, visited by field health worker, working status, distance to health facility, residence, region, household size, wealth index, ANC timing, frequency and facility

## Discussion

In this study, only one in ten women in Rwanda reported to have experienced good quality ANC. Of these, two thirds had initiated ANC within the first trimester, about half had 4 or more ANC contacts, and less than one percent had 8 or more ANC contacts. Exposure to newspapers/magazines, parity, being visited by a fieldworker, working status, household size, timing of first ANC visit, and frequency of ANC visits were associated with utilization of all components of ANC.

Although still very low, at 13.1%, this proportion of women who utilized all ANC components in Rwanda represents a significant increase from the 1.9% of the 2015 RDHS [12]. It is slightly higher than the East African region prevalence of 11% [13]. In 2019, Benova et al., analyzed DHS data from 10 LMICs’s, which showed that Rwanda’s current proportion of women receiving all ANC components is higher than Nigeria’s (6.7%), close to that of Democratic Republic of Congo (12.6%) and lower than that of Zambia (21.8%) [12]. However, the proportion is similar to that observed in a study based on Cameroon's most recent DHS data (13.5%) [26]. Although components such as having a blood sample taken, blood pressure measured, urine sample taken and receiving iron tablets/syrups were utilized by over 80% of the women (95.5%, 87.4%, 82.6% and 80.6% respectively), having at least two tetanus injections (33.6%) and receiving drugs for intestinal parasites (43%) were highly underutilized. A similar pattern of low tetanus toxoid vaccine among pregnant women has been registered in the East African region (50.4%) [35] and in The Gambia (34.8%) [36]. More efforts such as awareness and sensitization to women and increasing availability of immunization logistics and human resources are needed to ensure increased uptake.

Women visited by a fieldworker, were more likely to receive adequate ANC services compared to their counterparts that were not visited. Rwanda has a strong community health based programme as the lowest unit of its national health system [24]. Maternal health community health workers (CHWs) provide the first point of contact health services for most women seeking care [37]. Through a male–female CHW pair,women are able to receive the first line maternal and newborn health services within their own communities [20]. These CHWs ensure easier and equitable access to maternal care especially in hard-to-reach areas where health facilities are located far from the beneficiaries [37]. Interaction with field health workers has been documented to be associated with better maternal healthcare utilisation [37, 38]. This may be possible due to the counselling and information on key themes including the use of antenatal services, linkages to other reproductive health services, behavior change and delivery in health facilities [39, 40]. Furthermore, maternal health CHWs promote timely maternal health care seeking, document and follow up ANC appointments, promote male involvement in maternal health utilization and provide timely maternal health reports to their respective health centres with vital statistics such as number of pregnant women they have in their village, their ANC appointments and due dates [37, 41].

In this study, initiating ANC in the first trimester and attending ANC four or more times was associated with higher odds of receiving all components of ANC during pregnancy. Relatedly, women who made less than four ANC visits had lower odds of reporting quality ANC compared to those that had four or more visits. This is consistent with evidence from previous studies [42–44]. Initiating ANC in the first trimester accords opportunities to get timely ANC counselling and more time to have several ANC contacts in the subsequent months hence increasing the odds of receiving all the necessary components of ANC care [5, 42, 44]. Women who obtain 4 or more ANC visits have more contact with healthcare providers which renders them more likely to receive extensive health education and the best possible care [45, 46]. Invariably, 4 or more ANC attendance is an indication of a woman’s consciousness and commitment to her wellbeing in pregnancy [6, 26]. Additionally, frequent contact between the skilled provider and the pregnant woman helps in the development of good rapport between the two, enhances women’s familiarity with the health system, builds trust and confidence in the services received [47]. All these motivate women to freely share information with the ANC providers thereby facilitating the process of receiving more ANC components or reporting positively [2, 47, 48]. This demonstrates that Rwanda maternal health stakeholders need to prioritize explorative research to understand barriers of utilizing the recommended ANC contacts. These findings can help inform policy to ensure effective implementation of the latest WHO ANC model which recommends at least eight contacts for every pregnancy, in order to increase the odds of utilizing ANC components [42].

In this study, utilization of all ANC components reduced markedly with increasing parity. The odds of receiving all ANC components were six times higher among women who had at least one child, and reduced by half to three times higher among those with two to four children, compared to those with five or more children. This finding is not surprising because it has been reported consistently by studies in Ethiopia [45, 49], Haiti [50], Nigeria [51], and in an analysis of combined DHS data of six East African countries [13]. This could be due to the fact that women with multiple parities have a lower desire to continue attending all the recommended ANC visits. Largely because of the erroneous belief that they might no longer be in need of those services as they already have experience with pregnancy and childbirth [45, 49]. Therefore, they are less anxious and perceive their risk to be very low [13]. It could also possibly be because high parity pregnancies are more likely to be unintended, which has been linked to the later timing and lower amount of antenatal visits and hence reduced likelihood of receiving complete ANC components [52]. The increased odds of receiving all ANC components associated with low parity could also partly be linked to the observed association between women belonging to smaller households and good quality ANC. Women who belonged to smaller households had higher odds of receiving all ANC components compared to those from larger households a finding that has been shown in other studies [7, 53]. This might be partly attributed to availability of more resources and time because of less house chores in the smaller households [7, 52]. Having time enables women to initiate ANC early and have frequent subsequent ANC contacts hence leading to increased likelihood of receiving all the needed ANC contacts.

Working women had lower odds of receiving all ANC components. This is contrary to the evidence that women who work have higher prospects of effective maternal healthcare utilisation because they are likely to have a wider social network and receive information from other women they meet at the work place [6, 54, 55]. This may present an opportunity for them to learn about quality ANC and its importance and hence demand for it during ANC visits, unlike those who do not work. Similar to our findings, working women have been shown to have lower odds of utilizing quality ANC and other maternal health services [39, 56]. A possible explanation for this finding of a negative association could be related to the fact that working women do not have time to attend ANC care because of unfavorable work-related schedules and pressures such as limited or no leave days, and long working hours [39, 56, 57]. Subsequently, such barriers hinder their ability to initiate ANC on time and adhere to the recommended schedule of at least eight ANC contacts.

Exposure to newspapers/magazines was associated with higher odds of receiving all ANC components. Previous studies have showed a positive association between exposure to mass media and maternal health utilisation including ANC [58–61]. Exposure to mass media leads to positive health seeking behavioral changes through sharing information about the benefits of timely and frequent ANC contacts [3, 58]. Through mass media, women are also able to get information on availability of services and the working hours of these health facilities [39, 58, 62]. Furthermore, women that have access to newspapers are usually literate with better education, which allows them to engage in heath literacy related discussions with others. These discussions have the potential to challenge unfavorable stereotypes that may affect health seeking behaviors [5, 63, 64].

### Study strength and limitations

The study used a nationally representative dataset, making our findings generalizable to all women in Rwanda. In addition to considering the latest WHO ANC guidelines such as utilization of eight or more ANC contacts, the study used the most recent national data set with a large sample hence results are timely for policy makers to assess progress towards the global and national targets and implementation of the WHO ANC guidelines. However, the cross-sectional design doesn’t allow the establishment of causal relationships, but rather only associations. The use of self-reported answers without means of verification risked possibility of giving less accurate data. However, to mitigate this, data about the most recent birth was considered.

There was also a lack of data on other key determinants of adequate ANC such as male involvement and support, knowledge of ANC, the perceived quality/satisfaction of received care all of which could affect the uptake of ANC services and some health facility and health system determinants such as availability of medical supplies, waiting time etc. Lastly, the survey only included women with live births leaving out those with poor pregnancy outcomes such as still births.

Explorative qualitative research could be helpful in getting a deeper understanding why working women, those in Kigali and those belonging to larger households have lower odds of utilizing all ANC components. To enable comprehensive analysis of ANC and other maternal services in relation to the latest WHO guidelines, we recommend DHS to add more variables as per WHO guidelines recommendations such as use of ultrasound scan and specifying the timing and number of the ANC contacts in the different trimesters instead of just providing data on the timing of the first ANC contact. Furthermore, we recommend DHS to consider adding data on crucial factors such as male involvement and support, knowledge of ANC, the perceived quality/satisfaction of received care and some health facility and health system determinants such as availability of medical supplies, waiting time etc. or primary research that can incorporate the above factors.

## Conclusion

In Rwanda, the proportion of women who utilize all ANC components has improved since the last DHS, but it is still very low. Among the ANC components, TT injection uptake is at 33.6% which is the lowest hence more efforts such as awareness and sensitization to women, more research to explore factors associated with this low utilization and increasing availability of immunization logistics and human resources are needed to ensure increased uptake. Ministry of Health programs need to capitalize on existing positive interventions to further increase their coverage such as supporting women to subscribe to health insurance schemes and strengthening the community health programme to enable more field health workers providing counseling and providing care. High parous, unmarried, poor, Kigali resident women from larger households should be targeted for these programmes and policies aimed at increasing utilisation of ANC.

## Supplementary Information

**Additional file 1: Figure 1.** Flow chat of sampling process.

**Additional file 2.** Factors associated with ANC initiation timing and frequency as per RDHS 2019-20.

## Data Availability

The data set used is openly available upon permission from MEASURE DHS website (URL: https://www.dhsprogram.com/data/available-datasets.cfm).

## Abbreviations

EA: Enumeration area
AOR: Adjusted Odds Ratio
CI: Confidence Interval
COR: Crude Odds Ratio
DHS: Demographic Health Survey
RDHS: Rwanda Demographic Health Survey
OR: Odds Ratio
SD: Standard Deviation
WHO: World Health Organization
ANC: Antenatal care
PNC: Postnatal care
SBA: Skilled Birth Attendance
SPSS: Statistical Package for Social Science
