# Supplementary material for: Factors associated with utilization of quality antenatal care: a secondary data analysis of Rwandan Demographic Health Survey 2020
Source: BMC Health Serv Res. 2022 Jun 22;22:812. doi: 10.1186/s12913-022-08169-x (PMC9217119; doi:10.1186/s12913-022-08169-x)
Supplement: Supplementary file 1 — Additional file 1: Figure 1. Flow chat of sampling process. [file 12913_2022_8169_MOESM1_ESM.docx]

14,675 women aged 15 to 49 years eligible for interview

Excluded 41 not interviewed due to lack of consent or absent for the interview

14,634 women aged 15 to 49 years interviewed

8332 excluded since had no birth in the last 5 years not sampled for anthropometry

6302 had birth in the last 5 years prior to the survey

*Figure 1: flow chat of sampling process*
