# Supplementary material for: Factors associated with utilization of quality antenatal care: a secondary data analysis of Rwandan Demographic Health Survey 2020
Source: BMC Health Serv Res. 2022 Jun 22;22:812. doi: 10.1186/s12913-022-08169-x (PMC9217119; doi:10.1186/s12913-022-08169-x)
Supplement: Supplementary file 2 — Additional file 2. Factors associated with ANC initiation timing and frequency as per RDHS 2019-20. [file 12913_2022_8169_MOESM2_ESM.docx]

**Factors associated with ANC initiation timing and frequency as per RDHS 2019-20**

| **Characteristics** | **ANC timing**  **Adjusted model**  **aOR (95% CI)** | **ANC frequency**  **Adjusted model**  **aOR (95% CI)** |
| --- | --- | --- |
| **Age** |  |  |
| 35 to 49 | 1 | - |
| 20 to 34 | 0.90 (0.77-1.05) | - |
| 15 to 19 | **0.57 (0.38-0.86)** | - |
| **Education Level** |  |  |
| No Education | 1 | 1 |
| Primary Education | 0.99 (0.82-1.18) | 0.90 (0.74-1.09) |
| Secondary Education | 1.03 (0.80-1.31) | 0.86 (0.67-1.10) |
| Tertiary | **2.48 (1.50-4.10)** | 1.49 (0.95-2.34) |
| **Exposure to newspapers/magazines** |  |  |
| No | 1 | 1 |
| Less than once a week | 1.16 (0.96-1.39) | **1.20 (1.01-1.43)** |
| At least once a week | 1.40 (0.99-1.98) | **1.40 (1.06-1.86)** |
| **Exposure to radio** |  |  |
| No | 1 | 1 |
| Less than once a week | 1.17 (0.98-1.39) | 1.17 (0.98-1.39) |
| At least once a week | 1.07 (0.91-1.26) | 1.09 (0.92-1.29) |
| **Exposure to TV** |  |  |
| No | 1 | **1** |
| Less than once a week | 0.87 (0.74-1.01) | 0.88 (0.75-1.02) |
| At least once a week | 0.94 (0.75-1.18) | 0.99 (0.80-1.23) |
| **Internet access** |  |  |
| No | **1** | **1** |
| Yes | **1.54 (1.12-2.11)** | 1.33 (0.97-1.82) |
| **Parity** |  |  |
| 5 and above | **1** | **1** |
| 2-4 | **1.49 (1.21-1.84)** | **1.39 (1.15-1.68)** |
| Less than 2 | **1.77 (1.38-2.28)** | **1.53 (1.21-1.94)** |
| **Marital** |  |  |
| Not married | **1** | **1** |
| Married | **1.40 (1.19-1.65)** | **1.50 (1.28-1.76)** |
| **Has health insurance** |  |  |
| No | **1** | **1** |
| Yes | **1.27 (1.08-1.48)** | **1.67 (1.43-1.96)** |
| **Visited by a fieldworker** |  |  |
| No | **1** | **1** |
| Yes | **1.30 (1.15-1.46)** | **1.20 (1.07-1.35)** |
| **Working status** | **-** | **-** |
| Not working | **-** | **-** |
| Working | **-** | **-** |
| **Permission to access healthcare** |  |  |
| Big problem | **1** | **1** |
| Not big problem | 0.97 (0.71-1.34) | 1.19 (0.88-1.61) |
| **Distance to health facility** |  |  |
| Big problem | **1** | **1** |
| Not big problem | 1.10 (0.96-1.25) | 1.09 (0.95-1.24) |
| **Residence** | **-** | **-** |
| Rural | - | - |
| Urban | - | - |
| **Region** |  |  |
| North | 1 | 1 |
| East | 1.14 (0.94-1.38) | 1.02 (0.85-1.23) |
| West | 1.06 (0.86-1.30) | 1.12 (0.91-1.38) |
| South | 1.20 (0.99-1.47) | 1.18 (0.98-1.43) |
| Kigali | **0.56 (0.42-0.75)** | **0.76 (0.59-0.97)** |
| **Household Size** |  |  |
| 6 and above | 1 | 1 |
| Less than 6 | **1.29 (1.12-1.48)** | **1.16 (1.01-1.33)** |
| **Wealth Index** |  |  |
| Poorest | 1 | 1 |
| Poorer | 1.10 (0.92-1.32) | 1.18 (0.98-1.42) |
| Middle | **1.36 (1.12-1.66)** | **1.36 (1.12-1.65)** |
| Richer | **1.48 (1.20-1.83)** | **1.54 (1.25-1.89)** |
| Richest | **1.53 (1.13-2.08)** | **1.51 (1.16-1.97)** |
| **ANC timing** |  |  |
| After first trimester | - | - |
| Within first trimester | - | - |
| **ANC frequency** |  | - |
| Less than 4 | - | - |
| 4 and above | - | - |
| **ANC facility** |  | - |
| Private | - | - |
| Public | - | - |

**Bold**: significant at <0.05, ANC timing adjusted analysis included; age, education level, exposure to newspapers, radio and TV, internet access, parity, marital status, permission to access healthcare, distance to health facility, health insurance, visited by field health worker, region, household size and wealth index. ANC frequency adjusted analysis included; education level, exposure to newspapers, radio and TV, internet access, parity, marital status, permission to access healthcare, distance to health facility, health insurance, visited by field health worker, region, household size and wealth index.
